# The Integrative Taxonomy and Mitochondrial Genome Evolution of Freshwater Planarians (Platyhelminthes: Tricladida): The Discovery of a New Clade in Southern China

**DOI:** 10.3390/genes16060704

**Published:** 2025-06-13

**Authors:** Yimeng Yang, Zhizhuo Huang, Xiaowen Fang, Pinyi Li, Yexin Li, Xiuying Hou, Yongjun Li, Hengwen Yang, Chunxia Jing, Zhinan Yin, Guang Yang

**Affiliations:** 1 Department of Pathogen Biology, School of Medicine, Jinan University, Guangzhou 510632, China; ymyang924@163.com (Y.Y.); sparrowh@163.com (Z.H.);; 2 Department of Epidemiology, School of Medicine, Jinan University, Guangzhou 510632, China; 3 Key Laboratory of Viral Pathogenesis & Infection Prevention and Control (Jinan University), Ministry of Education, Guangzhou 510632, China; 4 The Biomedical Translational Research Institute, Faculty of Medical Science, Jinan University, Guangzhou 510632, China

**Keywords:** mitochondrial genome, molecular phylogeny, karyology, new species, planarian

## Abstract

Background: The genus *Dugesia* (Platyhelminthes: Tricladida) includes a large diversity of free-living freshwater flatworms and is important for studies on regeneration and evolution. This study aims to describe a newly discovered asexual planarian species from southern China and explore its genetic characteristics and regenerative abilities. Methods: An integrative taxonomic analysis was conducted using morphology, karyology, histology, molecular phylogeny (18S, 28S, COI, mitogenome), and genome size estimation via flow cytometry. Regeneration was assessed by standardized amputations, and long-term asexual propagation was observed under laboratory conditions for three years. Results: Phylogenetic analyses using nuclear (18S, 28S rDNA) and mitochondrial (COI, mitogenome) markers confirmed that *Dugesia cantonensis* Guang Yang & Zhinan Yin, sp. nov. forms a distinct clade within *Dugesia*. Its 18,125 bp mitogenome contains 36 genes but lacks atp8. *D. cantonensis* displays a distinctive morphology, notably a pharynx located near the head. All body fragments regenerated into complete individuals within nine days. Remarkably, one individual produced ~10⁵ clonal descendants over three years via repeated amputation, maintaining stable regenerative ability and growth across generations. Karyological analysis revealed a diploid karyotype (2*n* = 16) consisting of eight chromosome pairs. The nuclear genome size was estimated at approximately 2.5 Gb using *Danio rerio* as an internal standard. Histological examination showed no detectable reproductive organs, confirming the species as an exclusively asexual lineage. Conclusions: *D. cantonensis* represents a new planarian strain with stable propagation and regeneration. These features make it a valuable resource for regenerative biology and comparative genomic studies.

## 1. Introduction

The freshwater planarians of the genus *Dugesia* Girard, 1850, are the most primitive bilaterally symmetrical animals and occupy an important place in the history of species evolution [1]. Due to their undiminished regenerative ability, planarians have been chosen as one of the most appropriate experimental materials for stem cell research. About 100 species have been reported from most of the Old World and Australia [2]. However, for a long period after *Dugesia japonica* Ichikawa & Kawakatsu, 1964, was described from China, no new species of the genus were described, and it is only recently that taxonomic studies have begun to reveal the rich biodiversity of the genus in China [3,4,5,6]. Guangzhou is located in a subtropical coastal region, where the marine subtropical monsoon climate has fostered rich biodiversity. However, no Dugesiidae species have yet been recorded in this area. The discovery of a new species in Guangzhou would be an invaluable addition to the taxonomy of planarians in China. In this study, we aim to resolve the taxonomic challenges surrounding the evolutionary lineage and asexual strains of the Guangzhou planarian by establishing a localized strain and subsequently conducting investigations in regenerative biology, comparative genomics, and evolutionary biology.

Due to the great anatomical similarities between species, morphology-based phylogenetic analyses have struggled to resolve the affinities between species and species-groups [7]. The application of molecular markers offers a promising approach to overcome certain intrinsic constraints posed by morphological characteristics in species delineation and evolutionary history reconstruction among *Dugesia* [8]. *Dugesia* species exhibit a tripartite intestine, a T-shaped nervous system, and remarkable regenerative capabilities. Under suitable conditions, even small body fragments, including the head, tail, or trunk, can regenerate a fully functional organism.

The inclusion of karyological data may prove to be a highly informative additional line of evidence in systematic studies of the genus *Dugesia*. It has been reported that different *Dugesia* species exhibit differences in ploidy level and the centromeric position of the chromosomes [9,10,11]. Furthermore, several *Dugesia* species have been described with a chromosome portrait that differs from the most common haploid complement of n = 8, exhibiting complements such as *n* = 7 or *n* = 9 [12,13,14]. The results of chromosome karyotyping provide important reference value for understanding the evolutionary history of organisms and the kinship relationship between species.

The main characteristics of the mitochondrial genome as a very suitable phylogenetic marker include its small size, its abundance in tissues, the strict direct homology of the genes, the presence of genes or regions with different evolutionary rates, their uniparental inheritance, and the absence of recombination. In addition to the widely used gene sequences, other features of the mitochondrial genome can provide phylogenetic information, including gene content and rearrangement order, changes in the genetic code, or the secondary structure of tRNAs and rRNAs [15]. Internationally, the more well-studied *Schmidtea mediterranea* and *D. japonica* are markedly different in their morphological, nuclear, nuclear genomic, mitochondrial genomic, and transcriptomic data [16,17,18,19,20].

To further enrich our limited knowledge of the planarian species, we describe a new species of planarian from a region in southern China, a representative of the genus *Dugesia* Girard, 1850, on the basis of an integrative approach involving morphological, karyological, molecular data, and mitochondrial genome. The strain has already been successfully preserved and bred in the laboratory. According to the International Code of Zoological Nomenclature and the collection location, we named this new species as *Dugesia cantonensis* (Guang Yang & Zhinan Yin 2022) [21,22]. This study not only complements new molecular information about the family Dugesiidae in the order Tricladida but also provides a new experimental animal model for comparative research, aiming to enrich the molecular dataset of Dugesiidae species, elucidate their phylogenetic relationships within Tricladida, and establish a viable model organism for evolutionary and developmental studies.

## 2. Materials and Methods

### 2.1. Specimen Collection and Culturing

All specimens were collected, using a Pasteur pipette, from submerged leaves in an artificial freshwater pond in Guangzhou, Guangdong Province, China (23°10′48′′ N, 113°22′12′′ E), on 6 April 2022, and were identified based on their external morphology and light brown color pattern. The individuals were collected in a plastic bottle filled with habitat water during transport to the laboratory. The flatworms were maintained in Montjuïc water containing 1.6 mM NaCl, 1.0 mM CaCl_2_, 1.0 mM MgSO_4_, 0.1 mM MgCl_2_, 0.1 mM KCl, and 1.2 mM NaHCO_3_ in a 20 °C constant-temperature incubator in the dark. All animals were fed organic beef liver paste twice per week, and the culture solution was exchanged after each feeding. The flatworms were starved for 1 week before the experiments (being used in karyotype studies, growth curves, histological studies, or DNA extraction). The growth curves of planarians were created by recording the growth of planarians on days 0, 3, 6, and 9 using a digital camera. Thereafter, the body surface area was measured using Image J software version 1.53 and statistically analyzed using GraphPad Prism 9.5.1.

### 2.2. DNA Extraction, Amplification, Sequencing, and Phylogenetic Analysis

Total genomic DNA was extracted from 10 animals by using the E.Z.N.A. Mollusc DNA Isolation Kit (Omega, Norcross, GA, USA) according to the manufacturer’s protocols. Three gene fragments, 18S ribosomal gene (18S rDNA), 28S ribosomal gene (28S rDNA), and cytochrome C oxidase subunit I (COI), were amplified by polymerase chain reaction (PCR) with the Premix Taq (Takara, Osaka, Japan) according to the reference method [22,23,24]. The following primers were used: 18S rDNA (Forward primer: TACCTGGTTGATCCTGCC AGTAG, Reverse primer: GATCCTTCCGCAGGTTCACCTAC), 28S rDNA (Forward primer: GTCTTGAAACATGGACCAAGG, Reverse primer: GGAACCCCTTCTCCACTTCAGT), and COI (Forward primer: AGCTGCAGTTTTGGTTTTTTGGA, Reverse primer: ATGAGCAACAACATAATAAGTATCATG).

Forward and reverse DNA strands were determined by Sanger sequencing (IGE Biotechnology Co., Ltd., Guangzhou, China). The GenBank accession numbers of the species taxonomic sequences used in the phylogenetic analysis are shown in Supplementary Table S1. Sequence extraction and concatenation was performed using the software PhyloSuite v.1.2.3 [25]. The phylogenetic tree was constructed by using the maximum likelihood method with 1000 boosted replicates under the GTR + I + G model through Mega version 6 [26].

### 2.3. Mitochondrial Genome and Phylogenetic Analysis

The mitogenome of planarians was sequenced by the next-generation sequencing (NGS) method (Illumina Novaseq 6000, Shanghai, China). The quality of sequencing raw data was evaluated by FastQC and trimmed using Cutatapt software (Galaxy Version 1.16.6) [27]. Obtained Illumina reads were de novo assembled using the NOVOPlasty software version 4.3.1 [28], and the assembled genome was annotated by Mitos2 [29]. This NGS mitochondrial sequencing and linear assembling was completed by Shanghai Jinghan Biotechnology Co., Ltd. (Shanghai, China). We designed primers at both ends of the linear sequence to fill the gaps (F: GCAATTACTTTAGATGTTGCT, R: GCAGCATACATCCATTGCAG). The PCR program was set as follows: denaturation at 94 °C for 1 min, followed by 30 cycles of 98 °C for 10 s, annealing at 55 °C for 15 s, and extension at 68 °C for 5 min. The PCR-amplified products were electrophoresed on a 1% agarose gel, the purified PCR products were ligated with pUC18, and the positive clones were sequenced after transformation (Bio-Transduction Lab Co., Ltd., Shanghai, China). A complete cyclic mitochondrial sequence was finally obtained.

The secondary structures of mitochondrial transfer RNA (tRNA) genes were predicted by tRNAscan-SE (https://lowelab.ucsc.edu/tRNAscan-SE/, accessed on 26 July 2024) and MITOS (http://mitos2.bioinf.uni-leipzig.de/index.py, accessed on 26 July 2024). Circle maps of mitochondria were performed with Organellar Genome DRAW (OGDRAW) version 1.3.1 (https://chlorobox.mpimp-golm.mpg.de/OGDraw.html, accessed on 26 July 2024), The mitochondrial sequences were uploaded to the NCBI website and the mitochondrial circle diagrams were obtained according to the standard method [30].

Mitochondrial phylogenetic analysis was based on complete mitochondrial sequences of free-living flatworms annotated by the NCBI, but the outgroup here was *Prosthiostomum siphunculus* Delle Chiaje, 1822. Sequence extraction and concatenation were performed using the software PhyloSuite v.1.2.3 [25]. Multiple sequence alignments (MSAs) for protein-coding genes (PCGs) and ribosomal genes were carried out using MAFFT v7.505 [31], and the appropriate nucleotide substitution models were selected by trimming the compared sequences using the software trimAL (version 1.5.0) [32]. The best fit model for each PCG was selected by PartitionFinder2 [33]. Both the maximum likelihood (ML) method and Bayesian inference (BI) method used GTR + I + G models. ML analysis was conducted by IQ-TREE v1.6.2. For BI, MrBayes v3.2.6 was applied with 2,000,000 generations, sampling every 2000 generations, and the tree file was imported into the Interactive Tree of Life (iTOL) website (https://itol.embl.de/, accessed on 8 January 2025) for landscaping. The mitochondrial genome species used for mitochondrial analysis, along with the relevant GenBank accession numbers, are presented in Supplementary Table S2.

### 2.4. Karyotype Analysis and Nuclear DNA Content

The tail segments of the *D. cantonensis* (approximately 2.5 mm) were amputated and then incubated for 6h in the dark in a mixture of 0.25% colchicine and 1× Montjuïc water. The colchicine-treated tail segments were then placed in a Petri dish and rinsed with water. The tissue was then punctured with a needle in order to improve permeability, following which the tissue was incubated in deionized water for 20 min at room temperature. The tissue was then placed in fresh Carnot’s fixative (methanol–acetic acid 3:1 dilution) at 4 °C for 30 min. The tail fragment was then placed on a slide and soaked in 60% acetic acid for 5 min. The tissue was then flattened with a coverslip to form a monolayer of cells, after which they were incubated at 4 °C overnight. The slide was then removed and placed on ice, and a blade was used to quickly remove the coverslip. The slide was subsequently brought back to room temperature, and the procedure was repeated three times with 1x phosphate-buffered saline (PBS). Then, the slide was stained with a 1:5000 DAPI solution for 10 min and rinsed with PBS. Then, the slide was sealed and observed under a microscope [34].

Using flow cytometry to estimate the size of the nuclear genome, Zebrafish with known genomes were used as an internal reference species [35], standardized according to the kit provided by the manufacturer (Sysmex, 05-5022, Görlitz, Germany), and then the fluorescence intensity (Sysmex CyFlow^®^ Cube6, Singapore) was tested. The fluorescence intensity can represent the relative content of genomic DNA. The ratio of the DNA content of Zebrafish and flatworms can be derived from the fluorescence peaks of the tested sample and the PI-DNA complex of the Zebrafish sample, which can be multiplied by the C-value of the Zebrafish to calculate the C-value of the tested *D. cantonensis* [36]. *D. cantonensis* DNA content = [(*D. cantonensis* sample peak mean)/(Zebrafish standard peak mean)] × Zebrafish standard DNA content.

### 2.5. Histology

Following 7d of starvation, worms were euthanized using 5% HCl prior to fixation. After fixation in Bouin’s Fixative Solution (saturated nitric acid–formaldehyde–glacial acetic acid = 68:25:7), the worms were rinsed with 70% ethanol and then dehydrated in an ascending series of ethanol baths, cleared in terpineol, and embedded in wax (Paraplast Plus, Sigma, Saint Louis, MO, USA). Serial sections were prepared at 6 μm intervals and stained with hematoxylin and eosin. This was followed by visualization under a microscope (Leica, Wetzlar, Germany) [37]. Histological preparations of specimens have been deposited in the Laboratory of Department of Pathogen Biology, School of Medicine, Jinan University.

## 3. Results

### 3.1. Molecular Phylogeny of 18S rDNA, 28S rDNA, and COI

**Figure 1:**
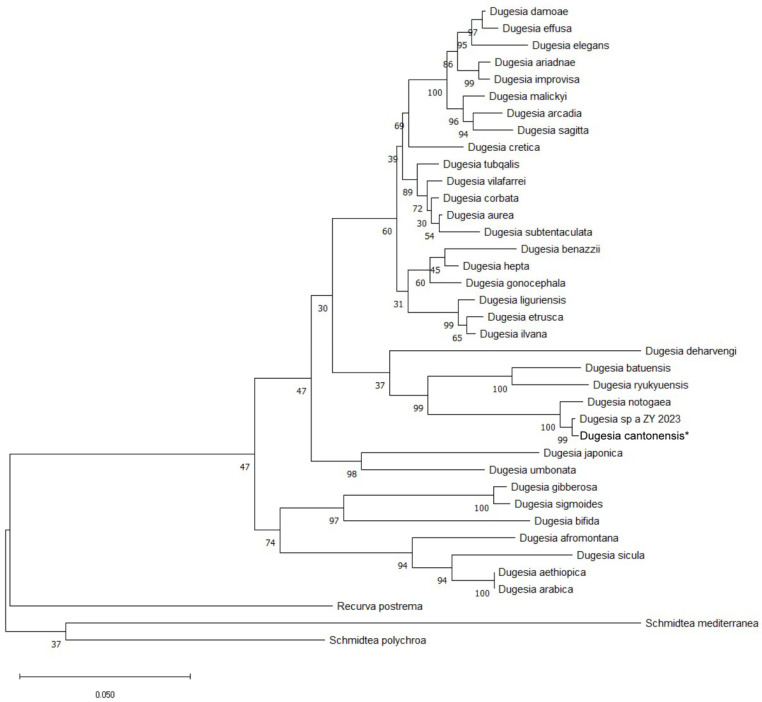
Maximum likelihood phylogenetic tree topology inferred from the concatenated dataset (18S rDNA, 28S rDNA, and COI) of representatives of the diverse species in the Dugesiidae family. Numbers at nodes indicate support values (bootstrap). Scale bar: substitutions per site. *: Represent our newly discovered species.

In order to identify the species of *D. cantonensis*, we used the maximum likelihood method to build a phylogenetic tree based on the concatenated nucleotide sequences of 18S rDNA (GenBank accession no. PQ901321), 28S rDNA (GenBank accession no. PQ901322), and COI (GenBank accession no. PV076738), and we ascertained the phylogenetic relationships among a total of 38 species within the family Dugesiidae. The phylogenetic tree showed a 30–100% level of support at all nodes. Within the broader taxonomic classification of the Dugesiidae family, *S. mediterranea*, *Schmidtea polychroa*, and *Recurva postrema* were chosen as the outgroup taxa [38]. The phylogenetic tree showed that *D. cantonensis*, *Dugesia* sp.a ZY 2023 (*D. ancroaria*), and *D. notogaea* form a clade. In this clade, the branch between *D. cantonensis* and *D. ancroaria* had support values (bootstrap) of 99%, and the common closest branch of *D. notogaea* had a value of 100% (Figure 1).

### 3.2. Mitochondrial Genomic Features and Phylogenetic Analyses

**Figure 2:**
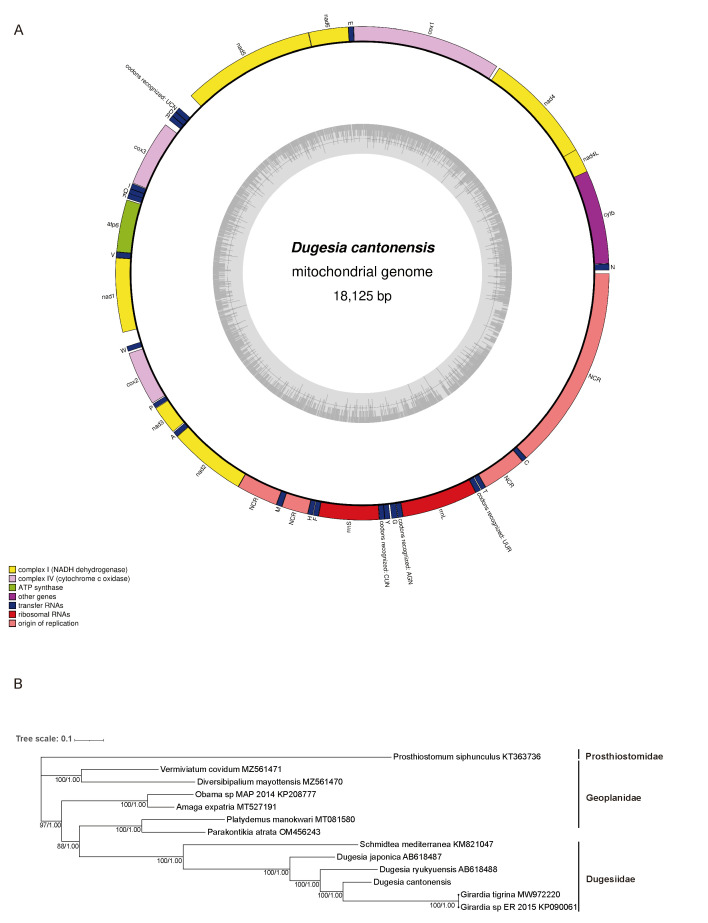
Map of the mitochondrial genome and phylogenetic relationships of *D. cantonensis* (GenBank accession no. PV083436). (**A**) Outer circle: different colors indicate different types of genes and regions. Inner circle: gray circle indicates GC content. Abbreviations: atp6, ATP synthase F0 subunit 6; cytb, Cytochrome b; cox1, Cytochrome c oxidase subunit I; cox2, Cytochrome c oxidase subunit II; cox3, Cytochrome c oxidase subunit III; nad1, NADH dehydrogenase subunit 1; nad2, NADH dehydrogenase subunit 2; nad3, NADH dehydrogenase subunit 3; nad4, NADH dehydrogenase subunit 4; nad4l, NADH dehydrogenase subunit 4L; nad5, NADH dehydrogenase subunit 5; nad6, NADH dehydrogenase subunit 6. (**B**) Phylogenetic tree obtained from the maximum likelihood and Bayesian analyses of the concatenated dataset for the protein-coding genes within mitochondrial genomes; the lines on the right represent different families; and the values indicated at the nodes are Bayesian posterior probabilities (right) and ML bootstrap proportions (left). Scale bar: substitutions per site.

In order to further ascertain the taxonomy of *D. cantonensis*, complete mitochondrial genome sequencing is carried out (GenBank accession no. PV083436). Its mitochondrial genome is 18,125 bp, which is a relatively larger size when compared with that of *S. mediterranea* at 17,176 bp (GenBank accession no. KM821047) and *D. japonica* at 17,799 bp (GenBank accession no. AB618487). The mitochondrial genome is composed of 36 genes (all genes are encoded in the same direction) consisting of 12 protein-coding genes, 22 tRNA genes, and 2 ribosomal RNA genes but lacking the subunit 8 of ATP synthase (atp8) gene, which is a common feature in flatworm mtDNA [39,40,41] (Figure 2A). The mitochondrial genome of *D. cantonensis* also exhibits deviations in base composition and skew (unequal representation of complementary bases on the same strand). Its composition is 23.3% A, 8.2% C, 15.8% G, and 52.6% T, and it is enriched in AT (A + T content of 75.9%), with T being the most common base on the coding strand. For the entire sequences, AT skew and GC skew are −0.31 and 0.32, respectively, when calculated using the formulae [42]. The lower value of AT skewness in these genes is generally considered to be associated with the formation of stem–loop secondary structures (i.e., base pairing between A and T in the stem regions of their corresponding secondary structures) [18,39].

The phylogenetic tree is constructed from concatenated protein-coding genes of the mitochondrial genome. The tree is composed of three families, Prosthiostomidae, Geoplanidae, and Dugesiidae of Platyhelminthes, which possess the annotation of a complete mitochondrial genome in NCBI. It uses *P. siphunculus* as an outgroup taxon. According to the phylogenetic tree, *D. cantonensis* belongs to the clade of the family Dugesiidae. The phylogenetic tree reveals that *D. cantonensis*, *D. ryukyuensis*, *Girardia tigrina*, and *Girardia* sp. constitute a clade, and this clade is comparatively close to *D. japonica* and *S. mediterranea*. The mitochondrial genome of *D. cantonensis* is more closely related to the *Girardia* genus, yet morphologically, its auricles are very different from those of the *Girardia* genus, which has pointed auricles (Figure 2B). The classification based on morphology and mitochondrial sequences clashes. We have given priority to the identification of specimens with obvious morphological differences [43].

### 3.3. Taxonomy

**Systematicaccount**
***Order Tricladida Lang, 1884***
***Suborder Continenticola Carranza, Littlewood, Clough, Ruiz-Trillo, Baguñà & Riutort, 1998***
***Family Dugesiidae Ball, 1974***
***Genus Dugesia Girard, 1850***
***D. cantonensis*** **Guang Yang & Zhinan Yin, sp.nov.2022**
**Zoobank registration:** urn: lsid: zoobank.org: pub: D04C1C71-0B3B-4EB5-95F3-9A40635090ED.
**Material examined:** an artificial freshwater pond, Guangzhou city, Guangdong Province, China, 23°10′48′′ N, 113°22′12′′ E, 6 April 2022, coll.Guang Yang and co-workers, sagittal section on 15 slides.
**Etymology:** The specific epithet *cantonensis* (Latin adjective, nominative singular masculine) originates from the historical exonym Canton (Guangzhou, China), combined with the Latin suffix -ensis (pertaining to). This nomenclature reflects the geographic origin of this species, which was first discovered in freshwater ecosystems of the Guangzhou region.
**Diagnosis:** *D. cantonensis* is characterized by a triangular head with a blunt, rounded tip accompanied by short, flattened auricles. The dorsal surface of the body has a light brown pigmentation with the body separated by a weakly pigmented dorsal midline stripe. The pharynx is located in the anterior of the body and is marked by a distinct white area. As a diploid organism, it possesses 16 chromosomes and a nuclear genome size of 2.5 Gb. The mitochondrial gene arrangement is as follows: cox1-E-nad6-nad5-S2-D-R-cox3-I-Q-K-atp6-V-nad1-W-cox2-P-nad3-A-nad2-M-H-F-rrnS-L1-Y-G-S1-rrnL-L2-T-C-N-cob-nad4l-nad4.

### 3.4. Description

**Figure 3:**
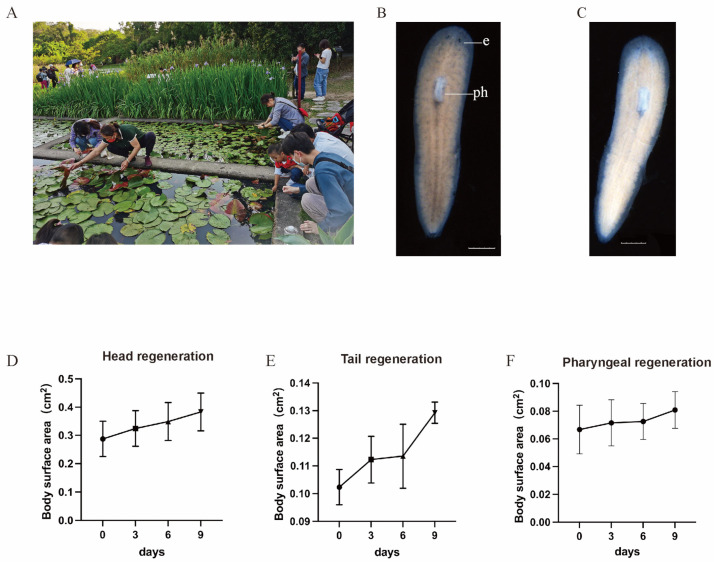
Habitat, external appearance, and regeneration of *D. cantonensis*. (**A**) Sampling site; (**B**) living asexual animal in dorsal view; (**C**) living asexual animal in ventral view; (**D**–**F**) regeneration rate of head, tail, and pharynx alone. The experiment was performed in triplicate (*n* = 20). e: eye spots; ph: pharynx. Scale bars: 1 mm.

*D. cantonensis* is elongated and constricted with a bluntly rounded anterior end. The posterior segment is a thin, conical tail end. Adult worms are about 1.0–1.2 cm long and light brown in color. The length of the pharynx is roughly one-tenth of the body length: the root position of the oropharyngeal is at around one-quarter of the anterior, while the oral opening is at approximately one-third of the anterior. The head and auricle are less pigmented. The two small eyespots are located near the anterior end and are oval in shape. The collection environment of *D. cantonensis* is shown in Figure 3A. There are no sexual organs in the posterior region (Figure 3B,C). In order to examine the regenerative capabilities of various body parts, *D. cantonensis* had their (trunk and tail) and head amputated (Figure 3D); planarians had their (head, trunk) and tail amputated (Figure 3E); and planarians had their trunk and (head and tail) amputated (Figure 3F). The whole regeneration process of the head, trunk, and tail resulted in complete regeneration by the 9th day. From a single *D. cantonensis*, it was propagated to approximately 100,000 individuals via amputation within 3 years, and tripling the time it stayed stable, suggesting the identification and nurturing of a new monoclonal planarian strain, *D. cantonensis*.

### 3.5. Karyology and Nuclear Genome Size Assay

**Figure 4:**
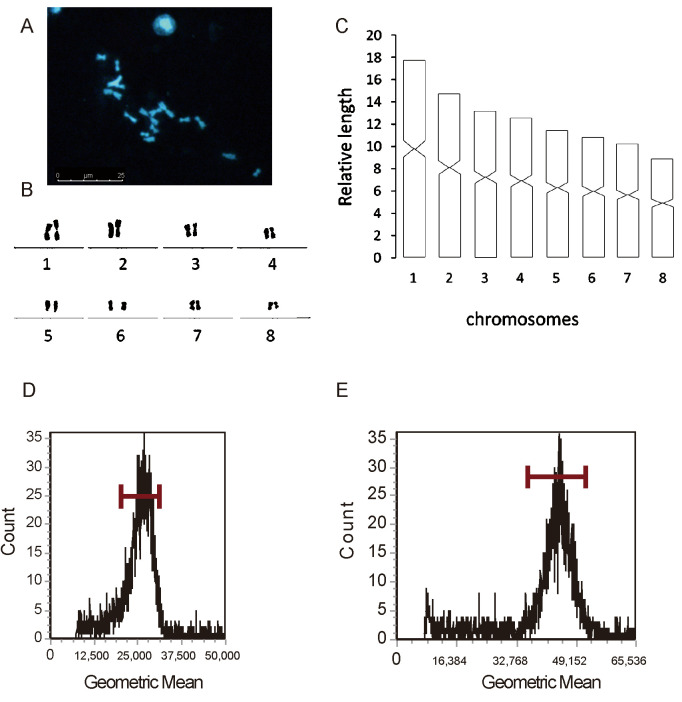
Metaphase spreads and absolute nuclear DNA amount (genome size) in *D. cantonensis*. (**A**) Microscope photo; (**B**) karyogram; (**C**) idiogram; (**D**) the Zebrafish (*Danio rerio*) serves as a reference species; (**E**) the relative DNA content of *D. cantonensis.* Scale bar: 25 µm.

In addition to molecular information, karyotype analysis and nuclear genome size are important complementary methods for species identification. Chromosomal measures and the population of individuals from the planarians were compiled [10]. As can be observed, the morphological structure of the chromosomes is obvious, with clearer mitoses, which facilitate karyotyping. *D. cantonensis* was diploid, with a chromosome complement of 2*n* = 16. Following the chromosome division criteria for determining the location of the chromosomes [44], chromosome 5 is found to be of the submetacentric type and the rest of the chromosomes are of the metacentric type. Karyotype parameters, including the relative length, arm ratio, and centromeric index, are given in Supplementary Table S3. A chromosomal plate and idiogram of the karyotype are shown in Figure 4A,C.

For nuclear genome size assay, the special fluorescent dye PI is used to bind to the DNA in the worm cells, and the cells dyed with the fluorescent dye emit fluorescence under laser irradiation. Utilizing the Zebrafish genome as an internal reference, which has an approximately 1.5 giga bp (Gb) haploid nuclear genome [45], the 2957 events were counted, with a gated cell rate of about 75.74% and a geometric mean of 26,167.11 in Zebrafish. *D. cantonensis* counted 3124 events with a gated cell rate of about 69.98% and a geometric mean value of 44,463,863. The results demonstrated that the size of the planarian genome is approximately 2.5 Gb. The size of the nuclear genome of *D. japonica* is 1.13 G and that of *S. mediterranea* is 773.9 Mb [17,19]. *D. cantonensis* has a relatively larger genome content with developmental complexity in its evolutionary position. In the phylum Platyhelminthes, the reduction in the nuclear genome is achieved by compression and gene loss as the organism moves from free-living to parasitic life [46], with the replication of smaller genomes or the transcription/translation of fewer genes requiring less energy, thus freeing up resources for other functions [47]. Variation in genome size can constrain, and simultaneously be constrained by, the evolution of the organism in which it finds itself [48] (Figure 4D,E).

### 3.6. Histology

**Figure 5:**
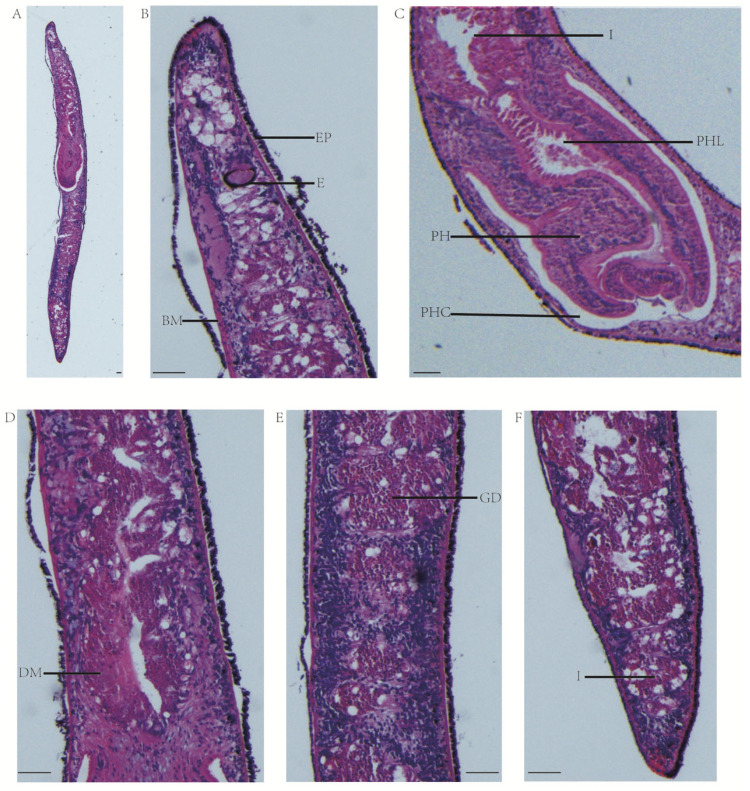
The sagittal sections of *D. cantonensis*. (**A**) HE staining of the sagittal plane of whole worm. (**B**–**F**) The sagittal structure of the planarian head, pharynx, posterior part of the pharynx, anterior part of the tail, and the tail. I, intestinal duct; PH, pharynx; PHC, pharyngeal cavity; PHL; pharyngeal lumen; (**E**) eye; EP, epidermis; DM, dorso-ventral muscle; GD, gastrodermis; BM, basement membrane. The scale bars: 75 μm.

To observe the organs of the planarian, histological examinations are carried out. After the worms have been stained, the structure of the organs is clearly visible (Figure 5A). In the head of the sagittal section, the structure of the eye point and the brain area can be clearly seen (Figure 5B). The structure of the planarian pharynx is clearly delineated, and above the pharynx is the intestinal duct. The entire pharynx is within the pharyngeal cavity and appears contracted. The pharyngeal lumen is located inside the pharynx (Figure 5C). The muscles and digestive tract are visible, but no reproductive structures such as seminal vesicle, copulatory bursa, vitelline gland, ejaculatory ducts, and oviducts are found (Figure 5D–F).

### 3.7. Protein-Coding Gene Preference Analysis and Transfer Ribosomal RNA of the Mitogenome

The Relative Synonymous Codon Usage (RSCU) value can measure the frequency of codon usage. As shown in Figure S1, the three codons with the higher RSCU values in the mitochondrial genomes of the *D. cantonensis* were UCU, GCU, and ACU, indicating that these three codons were the most frequently used in encoding the mitochondrial genome (Figure S1). The numbers on the bar graph represent the frequency of codon usage.

All 22 tRNAs were detected, with 21 exhibiting the typical cloverleaf structure. However, the circular structure of the glutamate tRNA was atypical, and the four stems characteristic of the cloverleaf structure were missing. This suggests that gene mutations may have occurred during the long-term evolutionary process. The secondary structures of the 22 tRNAs from the *D. cantonensis* newly sequenced mitogenomes were generated and are presented in Figure S2.

## 4. Discussion

The identification and nurturing of *D. cantonensis* represent a significant contribution to freshwater planarian biodiversity and evolutionary studies. The species exhibits unique morphological traits (e.g., pharyngeal position at the anterior quarter of the body), a larger mitochondrial genome (18,125 bp), a nuclear genome size of 2.5 Gb, and a diploid karyotype (2*n* = 16). These characteristics not only expand the taxonomic diversity of the genus *Dugesia*, but also provide a potential model for exploring genome evolution in free-living flatworms. The establishment of a stable culture stain facilitates research on regeneration, environmental adaptation, and developmental mechanisms.

The genus *Dugesia* exhibits a high degree of geographic population diversity on a global scale. This diversity is attributable to genetic differentiation resulting from geographic isolation, which in turn leads to the formation of new species of planarians [49,50]. However, the classification of freshwater planarians is challenging, and the limited availability of taxonomic keys to identify species, or the fact that most descriptions are old and based only on external morphological features, complicates the classification of some freshwater planarians [51]. The application of internal anatomical characteristics has resulted in alterations to the classification system over time [6]. In most instances, the absence of synapomorphies that define species or higher level taxonomic groups precludes the use of morphological features for the purpose of inferring phylogenetic relationships. Consequently, their classification is not based on their natural groupings as deduced from a phylogeny [52,53,54]. The use of molecular data to infer phylogenies has been pivotal for understanding the origin and evolution of numerous platyhelminth features, and molecular markers have become a vital tool for elucidating a plethora of diversity in many cases not, or only partially, predicted by morphological appearance [52]. This study utilized an integrative approach combining morphological, molecular, and cytogenetic data to validate *D. cantonensis* as a new species.

Nuclear genome sequencing remains critical but has not been fully explored, mainly because of its high cost and difficulty in assembly, especially in planarians, where few reference genomes are available and current taxonomic standards do not generally require nuclear genome data. However, nuclear genome data may increasingly complement traditional methods of species identification. The initial analysis at the higher level of taxonomy was conducted using 18S rRNA, with the subsequent addition of the mitochondrial gene COI, which has proven invaluable for intraspecific comparisons and for distinguishing closely related species [55,56]. In this study, we carried out phylogenetic analysis by means of complete mitochondrial sequences as well as 18S, 28S, and COI concatenated genes, and the results of the analyses were quite different from the evolutionary distances of *S. mediterranea* and *D. japonica*. The 18S, 28S, and COI exhibited high levels of saturation, which obscured the weak signals of other genes in the basal branches when working at the family level, as evidenced by the Continenticola phylogeny [24].

The complete mitochondrial genome offers a multitude of molecular markers that are conducive to the investigation of a variety of biological characteristics, including the impact of divergent ecological adaptations [57]. Moreover, the phylogenetic relationships among populations or species can be elucidated. This is because mitochondrial DNA usually does not recombine, frequently shows neutral evolution, and mt markers have smaller effective population sizes than their nuclear counterparts, which result in shorter coalescent times [58,59]. These features make mtDNA especially appropriate for either phylogeographical or population genetic studies [60]. In this study, we found that the size of the mitochondrial genome of *D. cantonensis* is relatively large when compared with that of *S. mediterranea*, *D. japonica*, and related *Dugesia ancoraria * [3].

Some freshwater planarians (Platyhelminthes, Turbellaria, Seriata, and Tricladida) switch between asexual and sexual reproduction [61,62]. The asexual individuals reproduce by fission without forming any sexual organs, whereas the sexual ones are hermaphroditic [63]. We sliced *D. cantonensis* by HE staining techniques. *D. cantonensis* have no obvious reproductive system in histology and are asexual worms. In a large number of observations regarding the behavioral science of planarians, the asexual species reproduces solely by fission and exhibits no sexuality whatsoever. Recent studies have shown that D-amino acids can be utilized to transform asexual worms into sexual worms [64]. Diploid organisms in *D. cantonensis*, which possess paired chromosomes, hold great potential in the shift towards sexuality. Gender transition is a scientific issue worthy of further study.

In addition, along with the progress of omics technologies, clarifying the biology of *D. cantonensis* from the perspectives of whole-genome sequencing, epigenomics, single-cell omics, metabolomics, and spatial transcriptomics will be advantageous and obtain more in-depth understandings of the development and pathogenesis of parasitic helminths.

Overall, we identified a new species of planarian and verified our findings by employing a range of techniques. The outcomes not only affirm the uniqueness of *D. cantonensis* but also provide insights into its evolutionary associations within the genus *Dugesia*. This discovery broadens the recorded diversity of planarians and presents a new strain for comparative studies.

## 5. Conclusions

In this study, we identify and describe a new asexual freshwater planarian species from southern China, *D. cantonensis*, using an integrative approach that combines morphological, karyotypic, molecular phylogenetic, and mitochondrial genomic analyses. The species displays distinctive morphological and genetic characteristics, exceptional regenerative capacity, and stable long-term asexual reproduction. Its mitochondrial and nuclear genomes suggest unique evolutionary and ecological adaptations. This discovery expands the known diversity of Dugesiidae in East Asia and offers a valuable model for research on regeneration, genome evolution, and asexuality.

## Data Availability

All sequences used in this study are available on GenBank.

## Supplementary Materials

The following supporting information can be downloaded at: https://www.mdpi.com/article/10.3390/genes16060704/s1, Table S1: GenBank accession numbers of sequences for species taxa used in the phylogenetic analyses; Table S2: Species and corresponding GenBank accession numbers of mitochondrial genomes used for mitochondrial analysis; Table S3: Karyotype parameters (mean values and standard deviations) of *D. cantonensis*; Figure S1: Base composition and relative synonymous codon usage (RSCU) values of *D. cantonensis*; Figure S2: Putative secondary structures of *D. cantonensis* mitochondrial genomes. The tRNAs are labeled with corresponding amino acid abbreviations.
