# Supplementary material for: The Integrative Taxonomy and Mitochondrial Genome Evolution of Freshwater Planarians (Platyhelminthes: Tricladida): The Discovery of a New Clade in Southern China
Source: Genes (Basel). 2025 Jun 13;16(6):704. doi: 10.3390/genes16060704 (PMC12192195; doi:10.3390/genes16060704)
Supplement: Supplementary file 1 [file genes-16-00704-s001.zip › Supplementary-Figure S1.pdf]

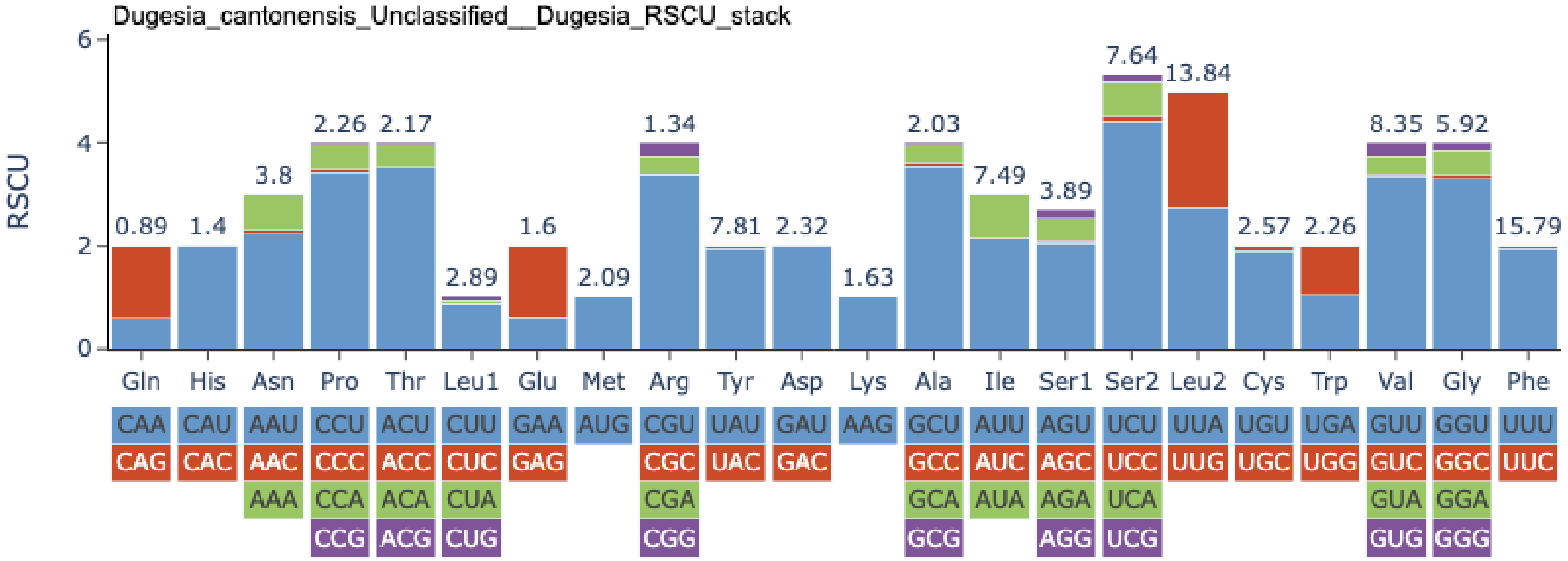

Figure S1. Base composition and relative synonymous codon usage (RSCU) values of *Dugesia cantonensis*.
