# Supplementary material for: The Integrative Taxonomy and Mitochondrial Genome Evolution of Freshwater Planarians (Platyhelminthes: Tricladida): The Discovery of a New Clade in Southern China
Source: Genes (Basel). 2025 Jun 13;16(6):704. doi: 10.3390/genes16060704 (PMC12192195; doi:10.3390/genes16060704)
Supplement: Supplementary file 1 [file genes-16-00704-s001.zip › Supplementary-Figure S2.pdf]

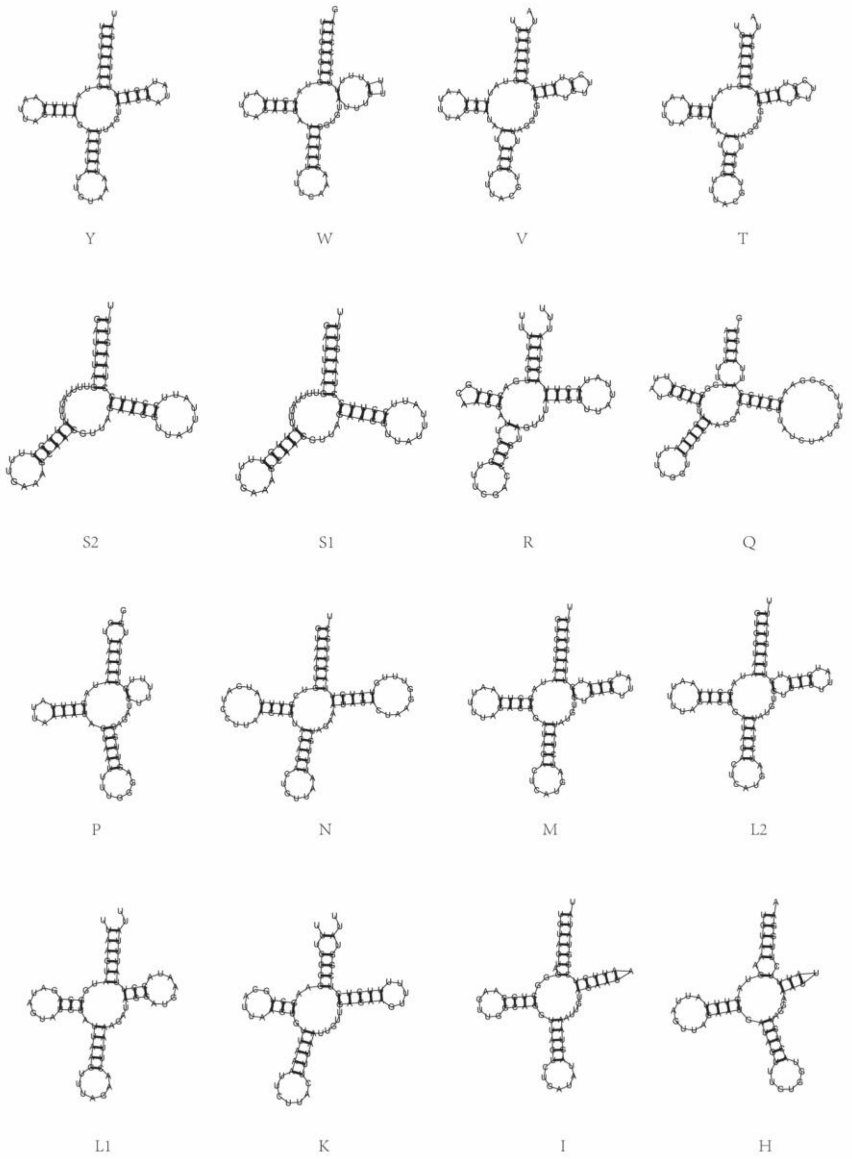
Y
W
V
T
S2
S1
R
Q
P
N
M
L2
L1
K
I
H

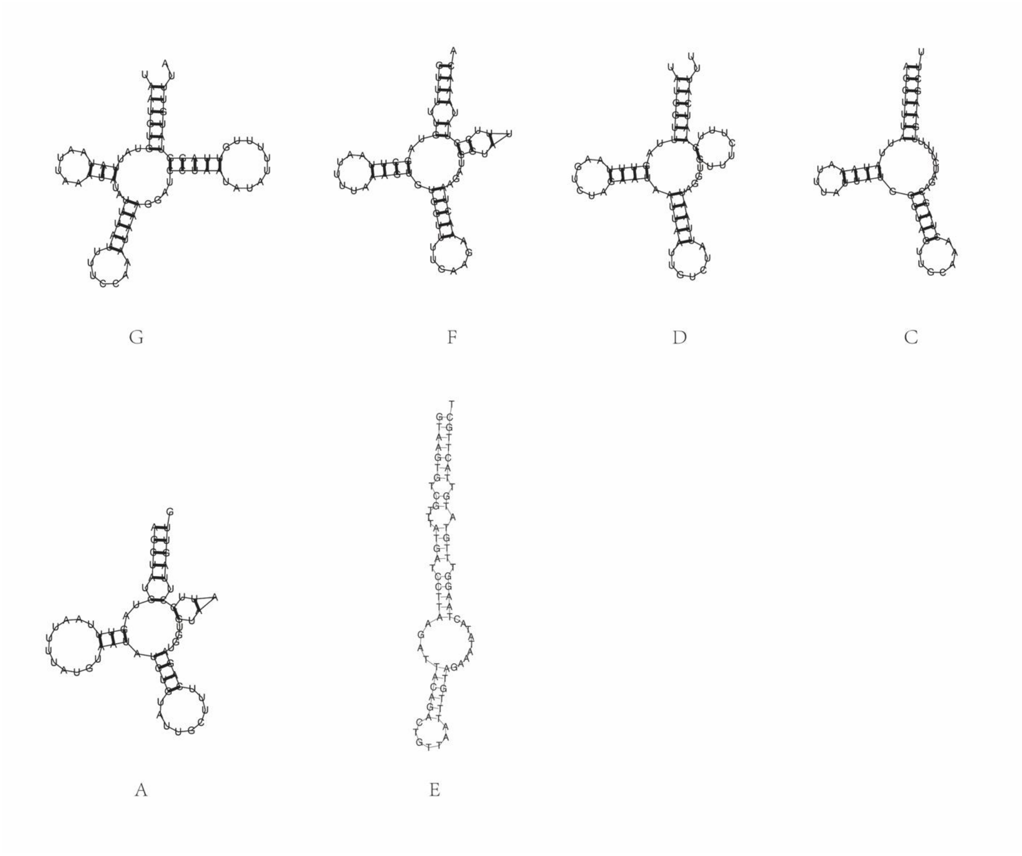

Figure S2. Putative secondary structures of *Dugesia cantonensis* mitochondrial genomes. The tRNAs are labeled with corresponding amino acid abbreviations.
