# Supplementary material for: The Integrative Taxonomy and Mitochondrial Genome Evolution of Freshwater Planarians (Platyhelminthes: Tricladida): The Discovery of a New Clade in Southern China
Source: Genes (Basel). 2025 Jun 13;16(6):704. doi: 10.3390/genes16060704 (PMC12192195; doi:10.3390/genes16060704)
Supplement: Supplementary file 1 [file genes-16-00704-s001.zip › Supplementary_Table_S1.pdf]

Supplementary Table S1. GenBank accession numbers of sequences for species taxa used in the phylogenetic analyses.

No.	species	18S	28S	COI
1	*D. aethiopica*	KY498822	KY498806	KY498845
2	*D. afromontana*	KY498823	KY498807	KY498846
3	*D. arabica*	OK646637	OK491342	/
4	*D. arcadia*	KF308694	OK491318	KC006971
5	*D. ariadnae*	OK646636	OK491317	KC006972
6	*D. aurea*	OM281823	MK712523	/
7	*D. batuensis*	OK646630	KF907821	KF907818
8	*D. benazzii*	/	OK491315	FJ646933
9	*D. bifida*	KY498843	/	KY498851
10	*D. corbata*	OM281826	MK712525	/
11	*D. cretica*	OK646620	OK491340	KC006976
12	*D. damoae*	OK646619	OK491310	/
13	*D. deharvengi*	/	KF907824	KF907820
14	*D. effusa*	OK646618	OK491311	/
15	*D. elegans*	KF308695	OK491313	KC006984
16	*D. etrusca*	OK646617	OK491312	FJ646939
17	*D. gibberosa*	KY498842	KY498819	KY498857
18	*D. gonocephala*	DQ666002	DQ665965	FJ646941
19	*D. hepta*	OK646612	OK491309	FJ646943
20	*D. ilvana*	OK646608	OK491334	/
21	*D. improvisa*	KF308696	OK491304	/

22	*D. japonica*	D83382	DQ665966	FJ646990
23	*D. liguriensis*	OK646615	OK491332	FJ646992
24	*D. malickyi*	OK646585	OK491294	/
25	*D. notogaea*	KJ599713	KJ599720	FJ646945
26	*D. ryukyuensis*	OK646571	OK491322	AF178311
27	*D. sagitta*	OK646567	OK491289	/
28	*D. sicula*	/	DQ665969	FJ646947
29	*D. sigmoides*	KY498827	KY498811	KY498849
30	*D. sp. a ZY-2023*	OR198141	OR225689	OR326966
31	*D. subtentaculata*	AF013155	MK712515	FJ646949
32	*D. tubqalis*	OK646555	OK491285	/
33	*D. umbonata*	MT177214	MT177210	MT176641
34	*D. vilafarrei*	OM281821	MK712511	/
35	*Recurva postrema*	KF308691	MG457274	KF308763
36	*S. mediterranea*	/	DQ665992	JF837062
37	*S. polychroa*	AF013152	/	FJ646975
38	*D. cantonensis**	PQ901321	PQ901322	PV076738
