# Supplementary material for: The Integrative Taxonomy and Mitochondrial Genome Evolution of Freshwater Planarians (Platyhelminthes: Tricladida): The Discovery of a New Clade in Southern China
Source: Genes (Basel). 2025 Jun 13;16(6):704. doi: 10.3390/genes16060704 (PMC12192195; doi:10.3390/genes16060704)
Supplement: Supplementary file 1 [file genes-16-00704-s001.zip › Supplementary_Table_S2.pdf]

Supplementary Table S2. Species and corresponding GenBank accession numbers of mitochondrial genomes used for mitochondrial analysis

Species	GenBank	Classification
Prosthiostomum siphunculus	KT363736	Platyhelminthes; Rhabditophora; Polycladida; Cotylea; Prosthiostomidae; Prosthiostomum.
Humbwetium covidum	MZ561471	Platyhelminthes; Rhabditophora; Seriata; Tricladida; Continenticola; Geoplanoidea;Geoplanidae; Bipaliinae; Vermiviatum.
Amaga expatria	MT527191	Platyhelminthes; Rhabditophora; Seriata; Tricladida; Continenticola; Geoplanoidea;Geoplanidae; Geoplaninae; Amaga.
Obama nungara	KP208777	Platyhelminthes; Rhabditophora; Seriata; Tricladida; Continenticola; Geoplanoidea;Geoplanidae; Geoplaninae; Obama.
Platydemus manokwari	MT081580	Platyhelminthes; Rhabditophora; Seriata; Tricladida; Continenticola; Geoplanoidea;Geoplanidae; Rhynchodeminae; Platydemus.
Diversibipalium mayottensis	MZ561470	Platyhelminthes; Rhabditophora; Seriata; Tricladida; Continenticola; Geoplanoidea;Geoplanidae; Bipaliinae; Diversibipalium.
Schmidtea mediterranea	KM821047	Platyhelminthes; Rhabditophora; Seriata; Tricladida; Continenticola; Geoplanoidea;Dugesiidae; Schmidtea.
Dugesia constrictiva	OK078614	Platyhelminthes; Rhabditophora; Seriata; Tricladida; Continenticola; Geoplanoidea;Dugesiidae; Dugesia.
Dugesia japonica	AB618487	Platyhelminthes; Rhabditophora; Seriata; Tricladida; Continenticola; Geoplanoidea;Dugesiidae; Dugesia.
Dugesia ryukyuensis	AB618488	Platyhelminthes; Rhabditophora; Seriata; Tricladida; Continenticola; Geoplanoidea;Dugesiidae; Dugesia.
Girardia tigrina	MW972220	Platyhelminthes; Rhabditophora; Seriata; Tricladida; Continenticola; Geoplanoidea;Dugesiidae; Girardia.
Girardia sp	KP090061	Platyhelminthes; Rhabditophora; Seriata; Tricladida; Continenticola; Geoplanoidea;Dugesiidae; Girardia.
Dugesia ancroaria	OR400685	Platyhelminthes; Rhabditophora; Seriata; Tricladida; Continenticola; Geoplanoidea;Dugesiidae; Dugesia.
*Dugesia cantonensis**	PV083436	Platyhelminthes; Rhabditophora; Seriata; Tricladida; Continenticola; Geoplanoidea;Dugesiidae; Dugesia.
