# Supplementary material for: The Integrative Taxonomy and Mitochondrial Genome Evolution of Freshwater Planarians (Platyhelminthes: Tricladida): The Discovery of a New Clade in Southern China
Source: Genes (Basel). 2025 Jun 13;16(6):704. doi: 10.3390/genes16060704 (PMC12192195; doi:10.3390/genes16060704)
Supplement: Supplementary file 1 [file genes-16-00704-s001.zip › Supplementary_Table_S3.pdf]

Supplementary Table S3. Karyotype parameters (mean values and standard deviations) of *Dugesia cantonensis*

**Chromosome**	**Relative length**	**Arm ratio**	**Centromeric index**	**Chromosome type**
1	8.22±0.23	1.13±0.04	47.04±0.85	metacentric
2	7.23±0.14	1.04±0.02	49.06±0.55	metacentric
3	5.70±0.17	1.40±0.07	41.65±1.28	metacentric
4	5.25±0.23	1.33±0.18	43.13±3.50	metacentric
5	5.14±0.08	2.75±0.11	26.67±0.78	submetacentric
6	4.17±0.20	1.41±0.16	41.58±2.77	metacentric
7	4.07±0.23	1.28±0.15	44.05±3.05	metacentric
8	3.60±0.20	1.11±0.02	47.38±0.55	metacentric
